# The Relationship between Selected Inflammation and Oxidative Stress Biomarkers and Carotid Intima-Media Thickness (IMT) Value in Youth with Type 1 Diabetes Co-Existing with Early Microvascular Complications

**DOI:** 10.3390/jcm11164732

**Published:** 2022-08-13

**Authors:** Joanna Peczyńska, Bożenna Klonowska, Beata Żelazowska-Rutkowska, Agnieszka Polkowska, Klaudyna Noiszewska, Artur Bossowski, Barbara Głowińska-Olszewska

**Affiliations:** 1 Department of Pediatrics, Endocrinology, Diabetology with Cardiology Division, University Children’s Clinical Hospital, Medical University of Bialystok, 15-274 Bialystok, Poland; 2 Department of Clinical Pediatrics, Faculty of Medical Sciences, Specialist Children’s Hospital, University of Warmia and Mazury in Olsztyn, 10-561 Olsztyn, Poland; 3 Department of Pediatrics Laboratory Diagnostics, University Children’s Clinical Hospital, Medical University of Bialystok, 15-274 Bialystok, Poland

**Keywords:** type 1 diabetes, macroangiopathy, IMT, biomarkers, inflammation, oxidative stress, microvascular complications, risk of cardiovascular disease, obesity, youth

## Abstract

Recent years have confirmed the importance of oxidative stress and biomarkers of inflammation in estimating the risk of cardiovascular disease (CVD) and explaining not fully understood pathogenesis of diabetic macroangiopathy. We aimed to analyze the relation between the intima-media thickness (IMT) of common carotid arteries and the occurrence of classical cardiovascular risk factors, together with the newly proposed biomarkers of CVD risk (high-sensitivity C-reactive protein (hsCRP), myeloperoxidase (MPO), adiponectin, N-terminal-pro B-type natriuretic peptide (NT-proBNP) and vitamin D) in youth with type 1 diabetes (T1D) recognized in screening tests to present early stages of microvascular complications (VC). The study group consisted of 50 adolescents and young adults with T1D, mean age 17.1 years (10–26 age range), including 20 patients with VC (+) and 30 VC (−). The control group (Control) consisted of 22 healthy volunteers, mean age 16.5 years (11–26 age range). In the VC (+) patients, we found a significantly higher concentration of HbA1c, lipid levels, hsCRP and NT-proBNP. BMI and blood pressure values were highest in the VC (+) group. Higher levels of MPO and lower levels of vitamin D were found in both diabetic groups vs. Control. IMT in VC (+) patients was significantly higher and correlated positively with HbA1c, hsCRP, NT-pro-BNP and negatively with vitamin D levels. In conclusion, youth with T1D and VC (+) present many abnormalities in the classical and new CVD biomarkers. hsCRP and MPO seem to be the most important markers for estimating the risk of macroangiopathy. NT-proBNP may present a possible marker of early myocardial injury in this population.

## 1. Introduction

According to the World Health Organization (WHO), cardiovascular disease (CVD) is now the first cause of death in most developed and many developing countries [1]. The atherosclerotic process, being the basis of CVD, begins already in early childhood, and its progression depends on the presence of identified disease risk factors [2]. Therefore, it is important to recognize and analyze the individual classical risk factors for cardiovascular disease and to take into account new biomarkers, which, in studies of the preclinical phase of the atherosclerotic process, seem to have a significant utility in estimating the risk of cardiovascular diseases and explaining the not fully understood pathogenesis of the disease [2].

Type 1 diabetes mellitus (T1D) is a chronic inflammatory illness, which results from a complete destruction of insulin-producing β-cells as an outcome of the combined action of autoreactive T lymphocytes, inflammatory cytokines and monocytic cells. Atherosclerosis as a primary cause of cardiovascular disease is a frequent, early and serious complication of T1D [3,4]. People with diabetes are two to four times more likely than others to develop CVD [5,6]. The estimated life expectancy is shorter by 14 years for women and even up to 17 years for men with the T1D onset during childhood [7]. Cardiovascular disease has overtaken diabetic kidney disease in being the leading reason for premature mortality in young adults with diabetes. The direction of disease complications prevention has been shifted to the age groups of the youth [8].

Many if not most diabetic children and adolescents do not have the optimal blood glucose control. Clinically apparent vascular complications associated with diabetes mellitus rarely occur at a young age [9]. However, early subclinical, mainly functional but also structural abnormalities, can occur a few years later after the disease onset, and they often intensify during puberty [9,10]. In about 8% of patients with T1D, increased urinary albumin excretion occurs after about 1–3 years of disease duration. After about 10 years, the albuminuria prevalence was reported to be 20% [11,12]. A 25% increase in GFR was found in children with T1D with disease duration of less than one year [13]. The first signs of non-proliferative retinopathy appear after about 4–5 years of disease duration [14]. Fortunately, there has been a declining incidence of complications reported in many recent research works. The incidence of retinopathy among T1D patients seems to diminish in longitudinal follow-up studies [15,16]. The lower incidence of retinopathy was associated with a decrease in HbA1c [17,18].

Over the previous decade, the atherosclerotic process was identified to be an inflammatory illness involving proinflammatory cytokines, and inflammation seems to play a crucial role in the pathophysiology of atherosclerosis [5]. Hyperglycemia-induced endothelial dysfunction, together with the increased oxidative stress and hypercoagulable potential of diabetes, accelerates the atherothrombotic complications process [19]. Oxidative stress is closely related to the pathogenesis of diabetes mellitus (DM) and comes as a result of overproduction of the reactive oxygen species (ROS) [19,20]. ROS overproduction is related to hyperglycemia and metabolic diseases, similar to the impaired antioxidant function [20,21]. In cases of DM, oxidative stress markers are overexpressed, suggesting that increased ROS can also be primarily responsible for the development of diabetic complications [22].

Hence, there has been a major focus of contemporary research on clinically useful markers to monitor the systemic inflammatory and oxidative stress burden and to search for a specific supportive therapy. The interplay between inflammation and oxidative stress in diabetes-accelerated atherosclerosis may be used to form a new concept in a practical way to envisage future cardiovascular risk by evaluating the inflammatory/oxidative stress biomarkers and designing clinical trials in which they are a therapeutic goal [18,23].

In recent years, the importance of the high-sensitivity C-reactive protein (hsCRP) determination by the ultra-sensitive method has been confirmed [24]. hsCRP is considered an independent predictor of the first cardiovascular incident, even stronger than classical lipid parameters [24,25,26]. The clinical utility of many other biomarkers is debated. Few oxidative status markers have been scientifically validated—myeloperoxidase (MPO) among them [27,28,29,30]. N-terminal-pro B-type natriuretic peptide (NT-proBNP), a useful biomarker of chronic heart failure, has turned out to be a strong indicator of cardiovascular mortality and a marker of atherosclerosis [31].

Studies conducted among young people using non-invasive ultrasound methods presented strong correlation between the abnormalities of the vascular structure and function and all classical CVD risk factors [32]. In many studies, it has been proven that the thickness of the medial and internal membranes of carotid arteries (intima-media thickness, IMT) is a sensitive indicator of coronary heart disease or stroke in patients with type 1 diabetes (DM1) [33].

Of note, studies in young patients suffering from type 1 diabetes mellitus and additional recognized early coexisting microangiopathy regarding CVD risk factors, new biomarkers and vascular status have not been conducted so far. The current problem faced by young patients with all chronic diseases, and especially T1D among them, is not only life expectancy but also quality of life, which largely depends on the condition of the cardiovascular system.

Therefore, the purpose of the presented study was to evaluate the cardiovascular risk factors and the chosen inflammation and oxidative stress biomarkers of atherosclerosis together with the assessment of IMT in young patients diagnosed with T1D and coexisting early microvascular complications recognized in screening tests. We intended to explain the issue of whether and how early microangiopathy in the type 1 diabetes course in young patients favors premature development of the atherosclerotic process and to find the most important related classical and new biomarkers that may predict early macroangiopathy. We assumed that the new knowledge may help create the appropriate therapeutic goals for these patients to minimize their cardiovascular risk and to better understand the mechanisms of atherosclerosis.

## 2. Materials and Methods

### 2.1. Patients

Adolescents and young adult patients with type 1 diabetes, of more than 5 years duration, aged more than 10 and under 26 years (considered the youth age group), mean 17.1 years, remaining under the standard care of the outpatient clinic of the Children’s Hospital in Olsztyn, were eligible for the study group. According to diagnosis, which confirmed or not the presence of microvascular complications, the recruited patients were allocated to the study groups: group 1 with diabetes without additional associated diseases T1D VC (−) (N = 30) and group 2 with diabetes and vascular complications T1D VC (+) (*N* = 20). Patients were classified into these specific groups on the basis of the results obtained at periodically performed screening tests, in accordance with the guidelines elaborated by the Polish Diabetes Society (PTD) and the International Society for Pediatric and Adolescent Diabetes (ISPAD). Additional diseases diagnoses were based on typical criteria presented in the Section 2.2 of the paper. The control group consisted of twenty-two healthy, age-matched peers—volunteers, mean age 16.5 years (Control). The protocol of the study was accepted by the Bioethics Committee of the Warmia and Mazury University, Physicians and Dentists Division in Olsztyn, Poland, IRB approval number: OIL 246/13/Bioethics (2013-06-04). In every case, the parents/guardians of a minor patient—and in the case of patient aged over 16 years, also the patients themselves—consented to participate in the study.

### 2.2. Methods

A Harpenden stadiometer and digital scale were used in a standard way for measuring the patients’ height and weight. Their body mass index (BMI) was calculated by a standard formula. The BMI-SDS, aiming to customize for age and sex, was calculated and assessed with the use of age- and sex-specific charts, on the basis of the local Polish OLAF study [34]. According to the BMI-SDS, we categorized patients as normal weight, overweight or obese. Their waist was checked with a centimeter measure and then calculated as SDS waist. In the study, we used two averaged measurements of systolic blood pressure and diastolic blood pressure from the patients’ right arm, each one performed after a 10 min rest with a calibrated sphygmomanometer.

Laboratory Analyses. The venous blood samples for laboratory tests were collected after an average 10 h of fasting (8–12 h) and centrifuged for 10 min at 2000 resolution per minute. HbA1c, lipid levels, vitamin D3, hormones and antibodies (thyroid—TSH, aTPO, ATG and celiac—the tissue transglutaminase IgA) were assessed by routine methods in the laboratory of the Children’s Hospital in Olsztyn, Poland. We used two ways to evaluate HbA1c: the last average value at the time the blood samples were taken and also the average mean value for the entire duration of the illness. Biomarkers—high-sensitivity C-reactive protein, adiponectin, natriuretic peptide—NT-pro BNP, myeloperoxidase (MPO) were determined in the Department of Pediatric Laboratory Diagnostics, University Children’s Teaching Hospital in Bialystok, Poland. hsCRP was figured out via the immunoturbidimetric method (Tina-quant hsCRP (Latex) HS, Roche). We analyzed adiponectin, natriuretic peptide and myeloperoxidase markers by using immunoenzymatic kits with the use of the ELISA method (R&D Systems, Inc., Minneapolis, MN, USA).

As microangiopathy, we defined the recognized disease of the microvessels/small blood vessels in the microcirculation. The diagnosis was made on the basis of ISPAD and PTD recommendations. We searched for nephropathy, retinopathy and neuropathy.

Albuminuria was estimated on the basis of the criteria of the Polish Diabetes Association. Albuminuria found at least twice in the period of 3–6 months with a value exceeding 30 mg albumin/day in a daily urine collection, was considered positive. Fourteen patients were diagnosed with persistent albuminuria; no patient was diagnosed with a more severe stage of nephropathy. Retinopathy was diagnosed on the basis of ophthalmoscopic examination, which was performed in the Ophthalmology Outpatient Clinic of the Regional Specialist Children’s Hospital in Olsztyn. Three patients were diagnosed with non-proliferative retinopathy where microaneurysms were proved, i.e., the first stage of diabetic retinopathy. The diagnosis of diabetic neuropathy was made on the basis of subjective symptoms and neurological examination, according to the current Polish Diabetes Society (PTD) recommendations and guidelines, performed in the Neurological Outpatient Clinic of the Provincial Specialist Children’s Hospital in Olsztyn [35]. Eight patients were diagnosed with peripheral, symmetric polyneuropathy, and three patients were diagnosed with autonomic cardiac neuropathy. Several patients were diagnosed with more than just one symptom of microangiopathy in different organs.

Ultrasound measurements. All children were examined in a quiet, temperature-controlled room. The procedure was conducted between 8.00 and 10.00 a.m. after a fasting period of 8–12 h. Examinations of the carotid arteries were performed by means of the apparatus of Hewlett Packard Sonos 4500, using a 7.5 MHz linear transducer. Measurements of intimal plus medial thickness in the common carotid arteries (right and left)—the value of IMT—were performed as previously described, with our own modification [36,37]. Briefly, the measurements included the end-diastolic (minimum diameter) IMT of the far walls (the distance from the leading edge of the first echogenic line to the leading edge of the second echogenic line), at the distance of more than 1 cm from the bifurcation. Analyses included the mean value of 6 measurements. No carotid plaques were found in any of the studied children.

Statistical Analyses. The continuous variables underwent tests for normal distribution. We used the Kolmogorov–Smirnov and Shapiro–Wilk tests. When variables met the criteria for normal distribution, the Student’s *t*-test was used. For the analyses of more than two groups, the variance analysis was used. Non-parametric Mann–Whitney U-tests for variables not meeting the normal distribution criteria were used to make a comparison between two groups. In the situation of comparisons for more groups, the median test and ANOVA rank Kruskal–Wallis test were applied. The results are shown as mean ± SD or median (Me) with the interquartile range. The univariate correlations analysis was performed with the use of the Spearman test. Finally, the multivariate regression analysis was performed to find the independent determinants of IMT. Variables for which the *p*-value in a single univariate analysis was <0.05 were included in this analysis. All comparisons were adjusted for gender, age, blood pressure and body mass index. The statistical analysis was conducted with the use of Statistica 13.0 (Stat Soft, Tulsa, OK, USA).

## 3. Results

We included fifty T1D patients, with mean age of 17.4 + 3.2 years, diabetes duration mean of 10.6 ± 3.01 years, HbA1c throughout the illness duration equal to 8.4 ± 1.3% and glycated hemoglobin at the time of the analysis of 8.74 ± 1.38%. The groups did not differ in mean duration of diabetes, age and daily insulin requirement. The reference group consisted of twenty-two (41% males) matched healthy peers. We present the general characteristics of the study groups in Table 1.

**Figure 1:**
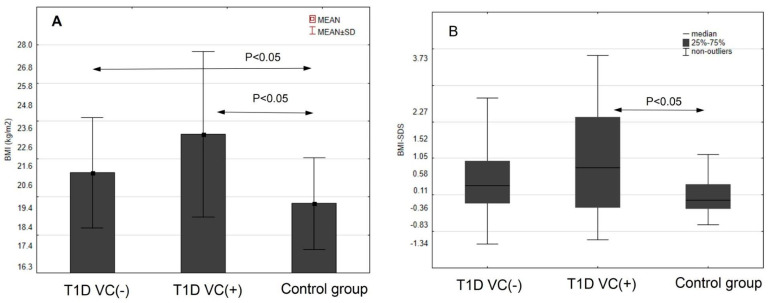

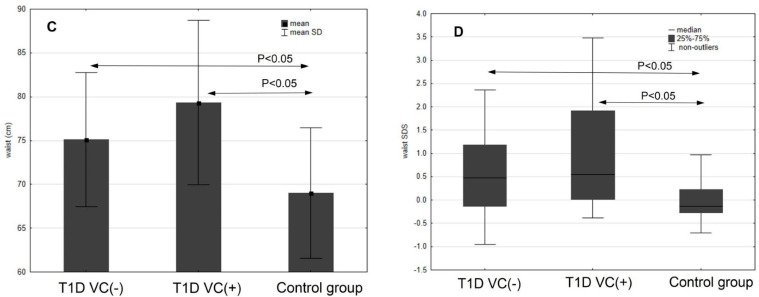
BMI (**A**), SDS-BMI (**B**), waist circumference (**C**) and waist SDS (**D**) in the studied groups.

At first, the traditional risk factors of cardiovascular complications were analyzed. In the variance analysis, all groups were compared one to another (Table 2, Figure 1). The highest BMI was found in group T1D VC (+) compared to the T1D (VC-) and to the reference group. Waist circumference was the highest in the group with T1D VC (+). Significantly higher standardized BMI-SDS and waist SDS were observed in the group with T1D and VC (+). There were statistically significant differences in both systolic and diastolic blood pressure (BP) values between the study groups. The highest mean systolic and diastolic BP was found in the group with T1D and VC (+). Lipids were most unfavorable in the group of diabetic patients with vascular complications, and as for triglycerides, we found a statistically significant difference. The last value of HbA1c and mean of the total disease duration were the highest in T1D VC (+) patients.

**Figure 2:**
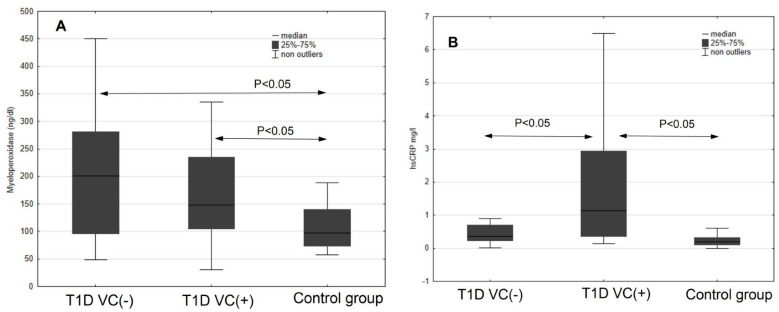

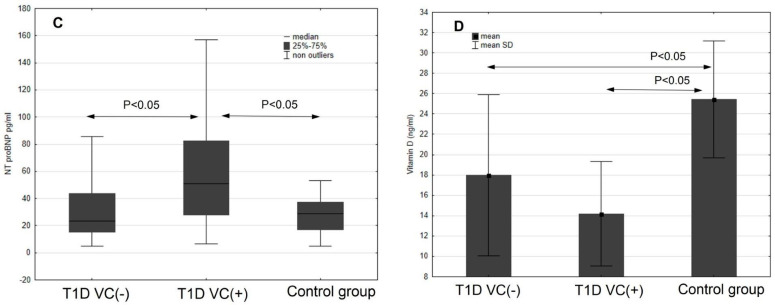
Selected “new biomarkers” (**A**–**D**) of the atherosclerotic process in the studied groups.

The results of new biomarkers of cardiovascular disease risk showed differences in the myeloperoxidase level (MPO), which was significantly higher in both diabetic groups compared to the healthy controls. More than five times higher levels of hsCRP protein, the highest concentration of NT-proBNP peptide and statistically lower concentration of vitamin D3 were found in the T1D VC (+) group. All patients with diabetes had lower mean vitamin D3 levels compared with controls. There were no statistically significant differences found for adiponectin in the study groups and the control group (Table 2, Figure 2).

**Figure 3:**
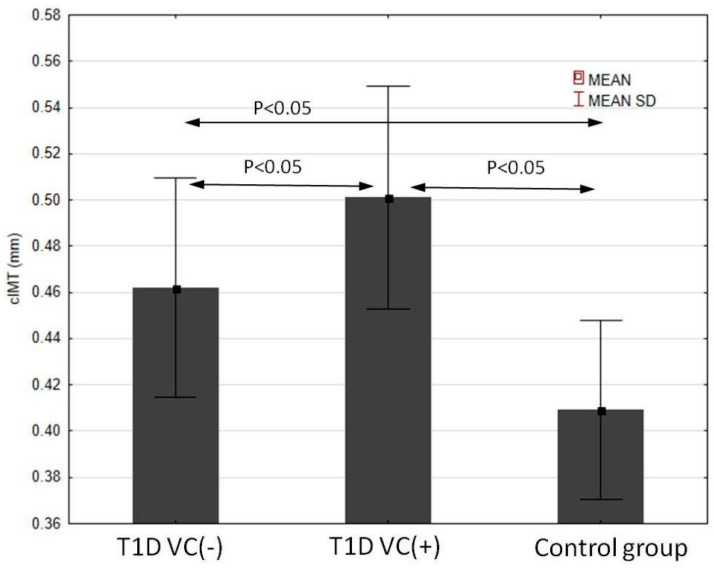
Carotid IMT in the studied groups.

**Figure 4:**
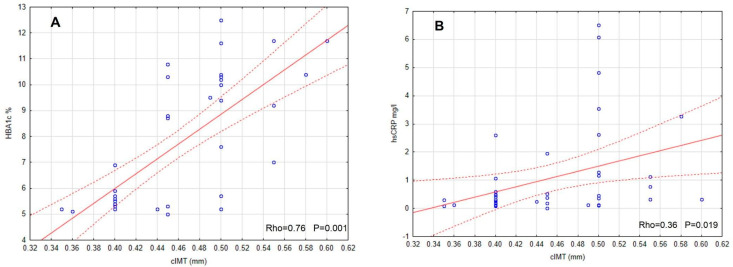

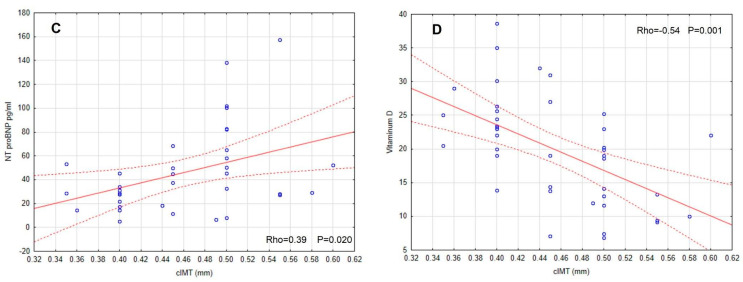
IMT correlations with chosen variables (**A**–**D**) in patients with T1D and vascular complications.

At the end, we investigated the IMT value. We found that the intima-media of the carotid arteries were significantly thicker in both diabetic groups in comparison to the controls. The IMT value was the highest in the group with T1D and VC (+), and the differences between the VC (−) group and the controls were significant (Figure 3). IMT correlated positively significantly with SDS-BMI, systolic blood pressure, HbA1c, hsCRP, NT-proBNP and inversely with vitamin D3 concentration (Figure 4). Table 3 shows other results of the correlations between intima-media thickness and the evaluated risk factors and the new biomarkers of CVD in T1D VC (+) patients. Multivariate regression analysis concerning these patients demonstrated that IMT was dependent on systolic blood pressure and glycated hemoglobin (R2 = 0.48, B = 0.18, *p* = 0.001).

## 4. Discussion

The main finding of our study is the significant impact of an adverse profile of classic cardiovascular risk factors in young patients (adolescents and young grown-ups) with T1D and coinciding microvascular complications (VC) compared with T1D without any additional disease. They represent an infrequently studied population of patients with diabetes. Cardiovascular risk factors are relatively generally well studied in complications-free diabetic children. Adult and older groups of patients with confirmed clinical complications and overt vascular disease are also well described. Our research provides an inimitable occasion to explore the status of cardiovascular risk factors in T1D patients who are nearly or only just adults, with a fairly long duration of diabetes but without clinically apparent vascular complications. Our current analysis showed significant differences in body weight, blood pressure and triglyceride values. Poorer metabolic control of diabetes, assessed as HbA1c over the entire disease duration and just the latest value, was confirmed in T1D and VC (+) patients. This group had higher BMI, SDS-BMI and waist circumference. This last indicator is crucial for the diagnosis of resistance to insulin in the clinical outcomes. Our findings provide further evidence that the VC (+) group of T1D had more altered parameters related to cardiovascular risk.

The accelerating frequency of worldwide obesity is evident in the growing number of cardiovascular conditions or cancers but also autoimmune disorders. Low-grade inflammatory process is recognized for the state of obesity, where numerous seditious cytokines and other markers are overproduced and overactivated. The increasing body weight has become a critical problem among patients with long-lasting T1D as well. Children weigh more at diabetes onset than some years ago, and their body mass increases with disease duration more than in their diabetes-free peers [38]. In a study assessing the young population with T1D from the United States, Germany and Austria, children and adolescents had higher BMI when compared to the reference values; twelve percent were recognized with obesity, twenty-four percent were found overweight [39]. BMI values exceeding the normal reference ranges were indeed found in every third child with T1D in another study [40]. The coincidence of excessive body weight with T1D is deliberated both as an environmental causative factor in the increased prevalence of T1D and as a result of the underlying disease itself [41]. Evans et al. reported that a high concentration of glucose and free fat acids generates oxidative stress and initiates insulin resistance (IR) in genetically predisposed individuals with diabetes [42,43]. Oxidative stress is the pathogenic element in diabetic endothelial dysfunction. Certain studies have evaluated the impact of obesity and overweight on the development of chronic complications in patients with T1D, suggesting that individuals with obesity are at a higher risk of macrovascular and some microvascular complications [44]. The development of central fatness, insulin resistance, inflammation and dyslipidemia, which are the main components of the metabolic syndrome and T2D and acknowledged risk factors of CVD, are associated with excessive weight gain in patients with T1D [45]. The coexistence of clinical characteristics of type 2 diabetes in patients with type 1 diabetes, such as insulin resistance and cardiovascular complications, was referred to as double diabetes [46]. This diabetes “subtype” is increasing in developed countries as a result of the outbreak of obesity among children and adolescents common in developed countries but exceedingly rare in developing countries [46,47].

Some authors have underlined that the presence of the metabolic syndrome in T1D should be noted as an independent risk factor for both macrovascular and microvascular complications [48]. We observed in the presented study that patients with an increased BMI also have a higher prevalence of risk factors—microvascular as well as macrovascular ones. Several observational studies of T1D have found connections between obesity, subclinical cardiovascular disease (CVD) [45,49,50] and mortality rates [51] within this population. Obesity increases mortality in T1D and could be a predictor of future cardiovascular events [52]. To sum up, the presence of overweight or obesity in young patients with T1D could have a major impact on serious metabolic consequences, particularly during adolescence.

Our current analyses proved that our diabetic groups represented poor metabolic control. HbA1c values deviated significantly from the recommendations; the poorest values were found in the T1D VC (+) group. This is a well-known clinical problem. It is veritably difficult for the youth to maintain the recommended metabolic control—indeed, even with use of ultramodern technologies [53]. It is highly possible that chronic hyperglycemia at this teenage age is the main contributor to IMT and microvascular complications [54]. The results of the SEARCH CVD Study stated, however, that the burden of cardiovascular risk factors gradually increased in youth with T1D, and body mass was a major modifiable risk factor in predicting increased IMT, and HbA1c alone could not clarify the value of IMT [55]. Yet, metabolic control, expressed as HbA1c, seems to be the most powerful CVD risk factor of arteriosclerosis. Recent DCCT/EDIC Study population research results revealed that HbA1c is correlated with numerous classical CVD risk factors, and its association cannot be treated as a clarification of its effect on cardiovascular risk. It is concluded that a multifactorial interaction of traditional nonglycemic CVD risk factors was indicated in all T1D cases. Thus, perfect metabolic control remains the primary goal [56].

The maintenance of glycemic target goals is of the utmost importance among all populations of diabetic patients, also among adults with type 2 diabetes and already clinically proven diabetic complications. In the recent elegant study by Badacz R, the authors aimed to investigate whether, in diabetic patients, the renal function (RF) and systolic (SBP) and diastolic blood pressure (DBP) values following stent-supported angioplasty (PTA) for atherosclerotic renal artery stenosis (ARAS) have an impact on cardiovascular and renal outcomes. In a group of 99 adult patients, they proved, among others, that major cardiac and cerebral events (MACCE) and progression to renal replacement therapy (RRT) were higher among those who did not reach the target glycemic goals compared to well-maintained T2DM at 24 months observation [57].

The risk of vascular complications development in type 1 diabetes in children, greater with worse metabolic control, is also exacerbated by lipid disorders [58]. The described results are in line with many formerly published research works considering youth diabetic cases [33,59]. Our studied T1D VC (+) group also presented an unfavorable lipid profile, in particular with elevated triglycerides.

The current results of the new cardiovascular risk biomarkers of our study showed activated oxidative stress, expressed by significantly higher myeloperoxidase levels in both diabetic groups, independently of the presence of complications. Most previous studies reported that myeloperoxidase is considered to play a very important role in the initiation and onset of cardiovascular disease, including by increasing LDL, oxidation and accelerating atherogenesis. According to Teng et al., children with T1D have significantly elevated plasma levels of myeloperoxidase, as well as the structural and functional changes associated with arteriosclerosis [60]. Elevated levels of this enzyme are a risk factor for the acute coronary syndrome [61,62].

An increased “low grade” inflammation state in our diabetic groups was present in our results, as proven by more than five times higher hsCRP protein levels compared to the control group. The relevance of hsCRP as the new and independent biomarker of CVD, associated substantially with increased body weight and adipose tissue and with low grade inflammation, is established [23,62]. Our study also verified that cases diagnosed with early microvascular complications had higher hsCRP levels in comparison not only to the healthy group but also to the T1D VC (−) group. Roughly 25 large studies based on observation published in the last thirty years have demonstrated high sensitivity of hsCRP, a biomarker of inflammation, as the independent predictor for CVD [63].

Only the T1D VC (+) group had significantly higher NT-proBNP peptide levels. Increased NT-proBNP levels are seen in heart failure, myocardial infarction and hypertension. In patients with acute coronary syndromes, NT-proBNP has an impact on prognosis [64,65]. In patients diagnosed with heart failure, NT-proBNP determination allows the monitoring of treatment and an assessment of its effectiveness. According to some authors, NT-proBNP is considered as one of the prognostic factors for the occurrence of cardiovascular events [31,66]. In patients with type 1 diabetes, with the preserved ejection fraction and without known heart disease, NT-proBNP was associated with increased risk of MACE and all-cause mortality [67]. With our results, we claim to speculate that increased NT-proBNP may be evidence of early impairment of the myocardial muscle and may serve as a marker of early development of heart failure in the future.

In our results, Vitamin D3 levels were significantly lower for both diabetic groups compared to controls. Some studies reported a reciprocal relation between the insufficiency of vitamin D and autoimmune disease risk [68]. Other studies showed lower vitamin D levels in overweight patients and ones with T1D [69,70]. Vitamin D supplementation was associated with improved blood vessel function in children with diabetes [70,71]. Whether vitamin D supplementation can be preventive in the general population or those likely to develop CVD is still open to debate [72,73].

Another interesting, crucial finding of our work was that the diabetic group with VC (+) had significantly higher IMT compared with VC (−) patients with T1D and the control. In the DMT1 VC (+) group, we found significantly positive correlations between IMT and BMI, systolic blood pressure, strong correlation with HbA1c value and negative correlation with vitamin D3. An association between leptin, adiponectin, hsCRP and IMT was found in youth with obesity in other studies [74,75]. Some publications report this functional intensive insulin treatment effect enhanced the control of glycemia and also significantly decreased the IMT index and prevalence of all vascular complications [45]. Recent observations showed that those from the intensive insulin therapy group who endured inordinate weight gain, after 15 years of observation, had increased intima-media thickness and CVD event prevalence compared to the group that was treated with the conventional method, thereby not gaining such good metabolic control. The increase in body mass in long-term observation appears to abate the success of the intensive insulin therapy treatment models and better metabolic control [1].

Our findings have potential therapeutic implications. Effective early prevention of cardiovascular disease before clinically apparent symptoms appear is becoming a major issue in pediatric diabetes care. With the increasing knowledge on mechanisms, which proved oxidative stress and inflammation to be involved in early atherosclerosis pathogenesis, new prevention and treatment strategies need to be developed and realized. They will include lifestyle modification and implementation of existing treatment guidelines. The recommendations indicate the necessity of using modern ultrafast acting insulin analogs, new diabetes technologies, namely insulin pumps with low glucose suspension function, and continuous glucose monitoring systems to improve metabolic control and reduce glucose variability. Recent treatment of obesity and insulin resistance in T1D with biguanides and treatment with new diabetes drugs typical for type 2 diabetes, such as GLP-1 analogs, SGLT-2 receptor inhibitors or even cases of bariatric surgery, have also been reported. The pharmacological treatment of hyperlipidemia and hypertension among young patients with T1D must be optimized. Large-scale prospective studies will be needed to determine the risks and benefits of early pharmacologic intervention in children and adolescents.

**Figure 5:**
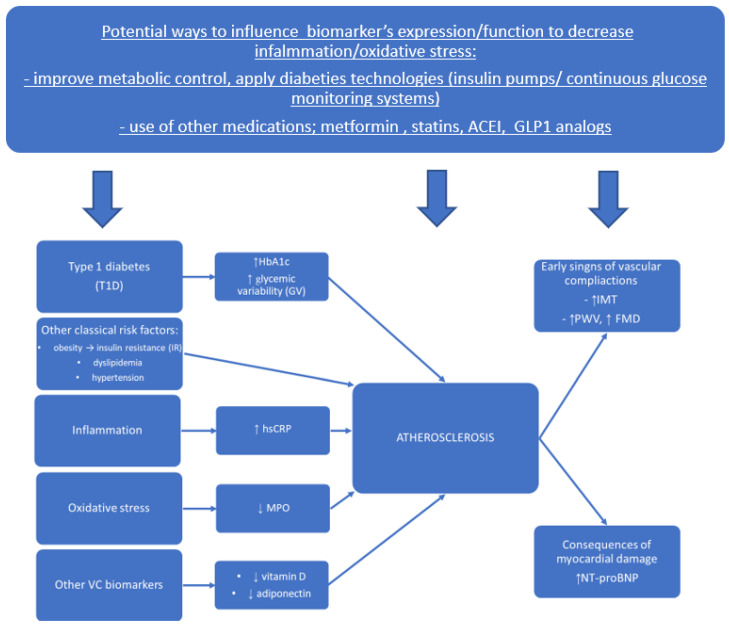
Proposed pathophysiological impact of classical risk factors and new biomarkers on the progression of atherosclerosis and possible ways to influence the process of diminishing vascular disease development.

The possible proposed pathophysiological impact of biomarkers on the progression of atherosclerosis and possible ways to influence the process of diminishing vascular disease development, based on our results and the papers cited in the Section 4, is presented in Figure 5.

## 5. Conclusions

Youth with type 1 diabetes with confirmed early microangiopathy present higher BMI, circumference of waist and HbA1c values and lipid alterations, which altogether can result in a premature development of diabetic cardiovascular complications in the near future. The extra risk of accelerated CVD may be a result of increased myeloperoxidase and CRP protein level, together with decreased vitamin D. Increased NT-proBNP may be evidence of early impairment of the myocardial muscle and a marker of early development of heart failure. The coexistence of microvascular complications significantly affected the IMT value in our T1D youth patients. Finding an explanation for whether and how additional early microvascular changes during the lifespan with type 1 diabetes mellitus may cause a premature atherosclerotic process can help identify the appropriate therapeutic targets for these patients to reduce their cardiovascular risk, but it may also be a further step toward understanding the mechanisms of inflammation and oxidative stress in atherosclerosis.

## Figures and Tables

**Table 1:** General characteristics of the study groups.

	T1D Total N = 50	T1D Group without Vascular Complications N = 30	T1D Group with Vascular Complications N = 20	Control Group N = 22
**Age (years)**	17.1 ± 3	16.8 ± 3.0	18.3 ± 3.3	16.5 ± 5.0
**Gender (M/F) [n(%)]**	20 (40%)/30 (60%)	13 (45%)/17 (57%)	10 (50%)/10 (50%)	9 (41%)/13 (59%)
**Diabetes duration (years)**	10.3 ± 3.1	10.0 ± 2.8	11.6 ± 3.2	
**Age of onset (years)**	6.8 ± 3.6	6.8 ± 3.9	6.7 ± 3.5	
**Body mass (kg)**	63.4 ± 14.5	60.3 ± 13.3	67.0 ± 14.0	54.0 ± 13.8
**Height (cm)**	170.6 ± 11	170.5 ± 11	169.4 ± 12	164.4 ± 13
**HbA_1_c mean (%)**	8.4 ± 1.3	8.1 ± 1.1	9.6 ± 1.2 *	
**HbA_1_c last (%)**	8.7 ± 1.2	8.6 ± 0.4	9.8 ± 1.6 *	5.4 ± 0.3
**Daily insulin requirement (UI/kg/24 h)**	0.8 ± 0.18	0.8 ± 0.16	0.8 ± 0.2	
**Remission period (months)**	7.9 ± 7.6	8.8 ± 7.6	5.6 ± 6.3	
**Creatinine (mg/dL)**	0.75 ± 0.16	0.8 ± 0.2	0.8 ± 0.2	0.8 ± 0.2
**AST (U/L)**	25.1 ± 18	21.6 ± 7.4	27.2 ± 12.1 *	23.5 ± 6.5
**ALT (U/L)**	28 ± 10	27.1 ± 10.0	32.8 ± 15.1	24.6 ± 7.1

The data are presented as mean ± SD. * *p* < 0.05 in Student’s *t*-test (difference between patients with T1D without vascular complications and T1D with vascular complications).

**Table 2:** Clinical characteristics, lipids, metabolic control, “new biomarkers” of cardiovascular disease between study groups. The results are presented as mean ± SD or median (interquartile range).

	T1D without Vascular Complications N = 30	T1D with Vascular Complications N = 20	Control Group N = 22	*p*-Values *
**BMI (kg/m^2^)**	^a^ 21.28 ± 2.9	^a^ 23.07 ± 4	19.65 ± 2.4	0.002
**BMI-SDS**	0.40 ± 1.0	^a^ 0.91 ± 1.5	−0.01 ± 0.58	0.021
**Waist (cm)**	^a^ 75.10 ± 7.6	^a^ 78.48 ± 9	69.0 ± 7.4	<0.001
**Waist SDS**	^a^ 0.54 ± 1.0	^a^ 0.93 ± 1.1	0.011 ± 0.79	0.009
**Systolic BP (mmHg)**	^a^ 121 ± 11	^a^ 129 ± 14	109 ± 9	<0.001
**Diastolic BP (mmHg)**	71 ± 6	^ab^ 77 ± 10	69 ± 5	0.003
**Total cholesterol (mg/dL)**	176 ± 25	^a^ 191 ± 38	164 ± 29	0.03
**LDL (mg/dL)**	101 ± 28	^a^ 112 ± 35	89 ± 28	0.06
**HDL (mg/dL)**	57 ± 11	^ab^ 52 ± 7	59 ± 11	0.05
**TG (mg/dL)**	82 ± 27	^ab^ 115 ± 67	75 ± 39	0.002
**HbA1c mean (%)**	8.1 ± 1.1	^b^ 9.6 ± 1.2	-	<0.001
**HbA1c last (%)**	^a^ 8.6 ± 0.4	^ab^ 9.8 ± 1.6	5.4 ± 0.2	<0.001
**Adiponectin (ng/mL)**	7704.6 (4816–10,231)	7279.8 (4541–11,926)	9746.6 (4933–11,333)	0.71
**Myeloperoxidase (ng/mL)**	^a^ 200.8 (95–281)	^a^ 147.750 (104–235)	96.8 (72–139)	0.06
**NTproBNP (pg/mL)**	23.4 (15.2–43.8)	^ab^ 51.07 (28–82)	28.9 (17–37)	0.01
**hsCRP (mg/L)**	0.36 (0.23–0.69)	^ab^ 1.14 (0.3–2)	0.2 (0.1–0.31)	<0.001
**Vit D (ng/mL)**	^a^ 17.9 ± 7.9	^a^ 14.3 ± 5	25.4 ± 5.7	<0.001

* ANOVA Kruskal–Wallis test; ^a^ *p* < 0.05—in comparison to the controls; ^b^ *p* < 0.05—in comparison to the diabetes group without vascular complication in post hoc analyses.

**Table 3:** Analysis of associations between IMT and studied variables in group of T1D with vascular complications VC (+).

	IMT	
**Age**	Rho = 0.1	*p* = 0.5
**Diabetes duration**	Rho = −0.22	*p* = 0.33
**BMI**	Rho = 0.35	*p* = 0.03
**BMI-SDS**	Rho = 0.22	*p* = 0.15
**Waist SDS**	Rho = 0.2	*p* = 0.2
**Systolic BP**	Rho = 0.58	*p* < 0.001
**Diastolic BP**	Rho = 0.26	*p* = 0.084
**Total cholesterol**	Rho = 0.11	*p* = 0.45
**LDL**	Rho = 0.19	*p* = 0.21
**HDL**	Rho = −0.42	*p* = 0.004
**TG**	Rho = 0.26	*p* = 0.087
**HbA1c mean**	Rho = 0.76	*p* = 0.001
**Adiponectin**	Rho = 0.23	*p* = 0.19
**Myeloperoxidase**	Rho = 0.12	*p* = 0.51
**NT-proBNP**	Rho = 0.39	*p* = 0.020
**hsCRP**	Rho = 0.36	*p* = 0.019
**Vit D**	Rho = −0.54	*p* = 0.001

## Data Availability

The raw data supporting the conclusions of this article will be made available by the authors, without undue reservation.
